# Current and experimental therapeutics for Fabry disease

**DOI:** 10.1111/cge.13999

**Published:** 2021-05-25

**Authors:** Vanessa Castelli, Cosimo Andrea Stamerra, Michele d'Angelo, Annamaria Cimini, Claudio Ferri

**Affiliations:** ^1^ Department of Life, Health and Environmental Sciences University of L'Aquila L'Aquila Italy; ^2^ Sbarro Institute for Cancer Research and Molecular Medicine, Department of Biology Temple University Philadelphia Pennsylvania USA

**Keywords:** enzyme replacement therapy, Fabry disease, in vivo models, in vitro models, lysosomal storage disorder, therapeutic approaches

## Abstract

Fabry (or Anderson‐Fabry) is a rare pan‐ethnic disease affecting males and females. Fabry is an X‐linked lysosomal storage disease, affecting glycosphingolipid metabolism, that is caused by mutations of the *GLA* gene that codes for α‐galactosidase A. Fabry disease (FD) can be classified into a severe, classical phenotype, most often seen in men with no residual enzyme activity, that usually appear before 18 years and a usually milder, nonclassical (later‐onset) phenotype that usually appear above 18 years. Affected patients show multifactorial complications, including renal failure, cardiovascular problems, and neuropathy. In this review, we briefly report the clinical trials so far performed with the available therapies, and then we focus on the in vitro and the in vivo experimental models of the disease, to highlight the relevance in improving the existing therapeutics and understand the mechanism of this rare disorder. Current available in vivo and in vitro models can assist in better comprehension of the pathogenesis and underlying mechanisms of FD, thus the existing therapeutic approaches can be optimized, and new options can be developed.

## Graphical abstract

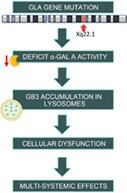

## 1 INTRODUCTION

**FIGURE 1:**
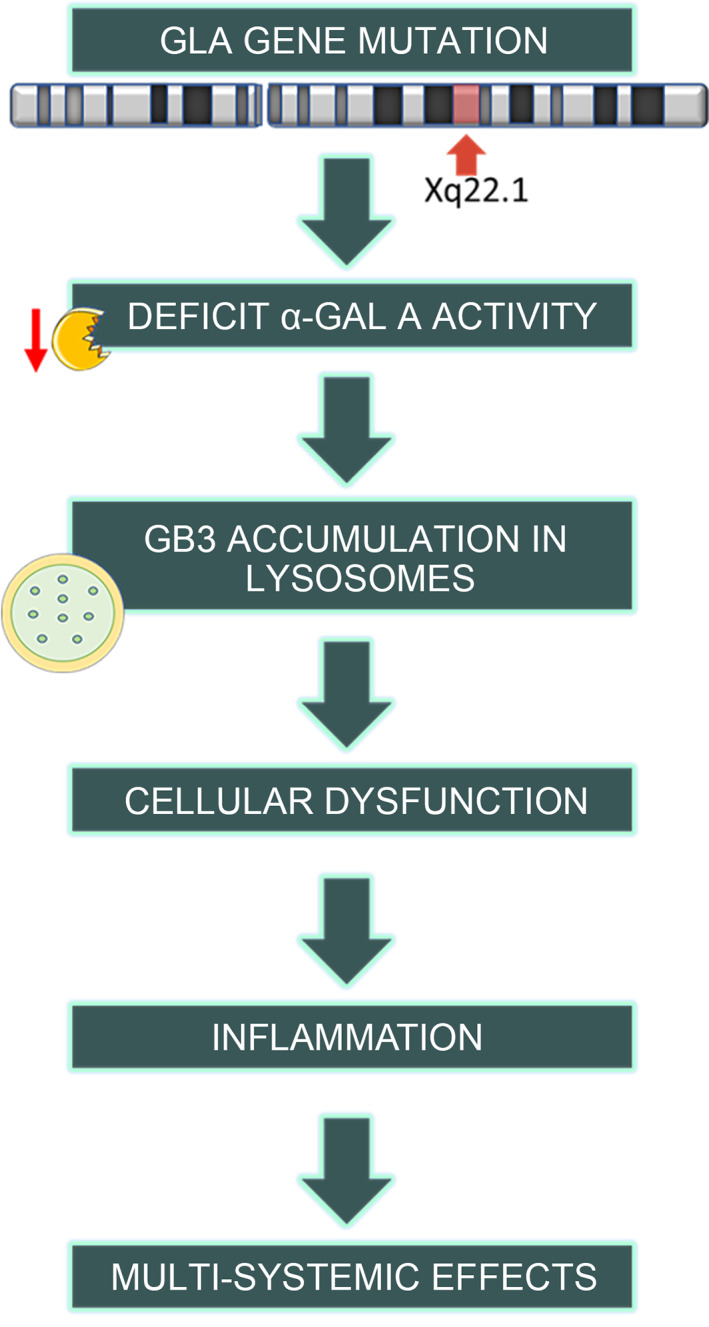
Schematic representation of the pathogenic mechanism that occurs in Fabry disease. GB3, globotriaosylceramide; GLA, Alpha‐Galactosidase A

Fabry disease (FD, OMIM #301500) is an X‐linked lysosomal storage disorder, involving glycosphingolipid metabolism. FD is due to a wide range of mutations in the *GLA* gene on the X chromosome (Xq22.1), resultant in a defect of the lysosomal enzyme α‐galactosidase A (*α‐gal A*).^1^ This induces to gradual deposits of globotriaosylceramide (Gb3) in cells in whole body, producing multi‐systemic impacts, including serious and progressive cardiac and renal impairment^2^ (Figure 1). Gb3 and its metabolite, lyso‐Gb3, are crucial in the pathogenesis of FD. Lyso‐Gb3 is produced by the deacylation of Gb3 by acid ceramidase.^3^ Lyso‐Gb3 accumulation worsens the disease pathology both reducing *α‐gal A* activity and promoting smooth muscle cells proliferation,^4^ an element that may promote the increased intima‐media thickness observed in Fabry patients.^5^ Lyso‐Gb3 destroys nociceptive neurons, leading to kidney fibrosis and inhibiting endothelial nitric oxide synthase.^6^Lyso‐Gb3 is strongly increased in plasma of classical FD male patients as well as in tissues and plasma of mice, but it does not correlate with the severity of the disease and cannot represent a valid surrogate biomarker.^7^, ^8^

*α‐gal A* deficiency induces the activation of numerous pathways and impacts the onset of disease symptoms. Downstream effects, including inflammation, fibrosis, and the reactive oxygen species production also appear to exert a role in the pathogenesis.^9^, ^10^

Fabry is a multisystemic disorder that initiates at cellular level (disruption of basic metabolic processes and a cascade of events), followed by a cascade of organ dysfunctions and structural alterations that ultimately progresses over years or decades. The age of onset is widely variable as well as clinical manifestations and progression. The primary disease begins as early as the fetal phase of development.^11^ FD can be classified into a severe, classical phenotype, most often seen in men with no residual enzyme activity, that usually appear before 18 years; and a usually milder, nonclassical (later‐onset) phenotype that usually appear above 18 years.^12^

The first clinical manifestations in FD classical phenotype occur typically between 3 and 10 years of age with features in boys more severely than girls.^13^, ^14^ The most frequent sign in classical Fabry hemizygous males, with no residual *α‐gal A* activity, are cornea verticillata, neuropathic pain, cardiomyopathy, strokes (cerebrovascular), angiokeratoma, proteinuria, arrhythmia, cochleo‐vestibular, renal and gastrointestinal disorders.^15^ Renal, cerebrovascular and cardiac disorders are manifested after the age of 20 years.

In later‐onset FD patients, the symptoms are milder or may be limited to one organ since they have residual *α‐gal A* activity (5–25% of normal) and have a later onset (average age: 40–60 years).^12^, ^16^ Long–term clinical manifestations comprise progressive renal failure, stroke, and hypertrophic cardiomyopathy.^12^ Women often present signs and symptoms of FD, although less severe compared with men.^17^ Fortunately, the knowledge about FD natural history and potential therapeutic approaches are unceasingly evolving and consequently the recommended testing, treatment procedures and monitoring need to be revised.

In this review, we report the current and experimental therapeutic approaches for FD. In addition, we focus on the in vivo and in vitro models of the disease, which are valuable for dissecting the pathogenesis and mechanism of FD and for optimizing the existing therapeutics or developing new options.

## 2 THERAPEUTIC APPROACHES

**FIGURE 2:**
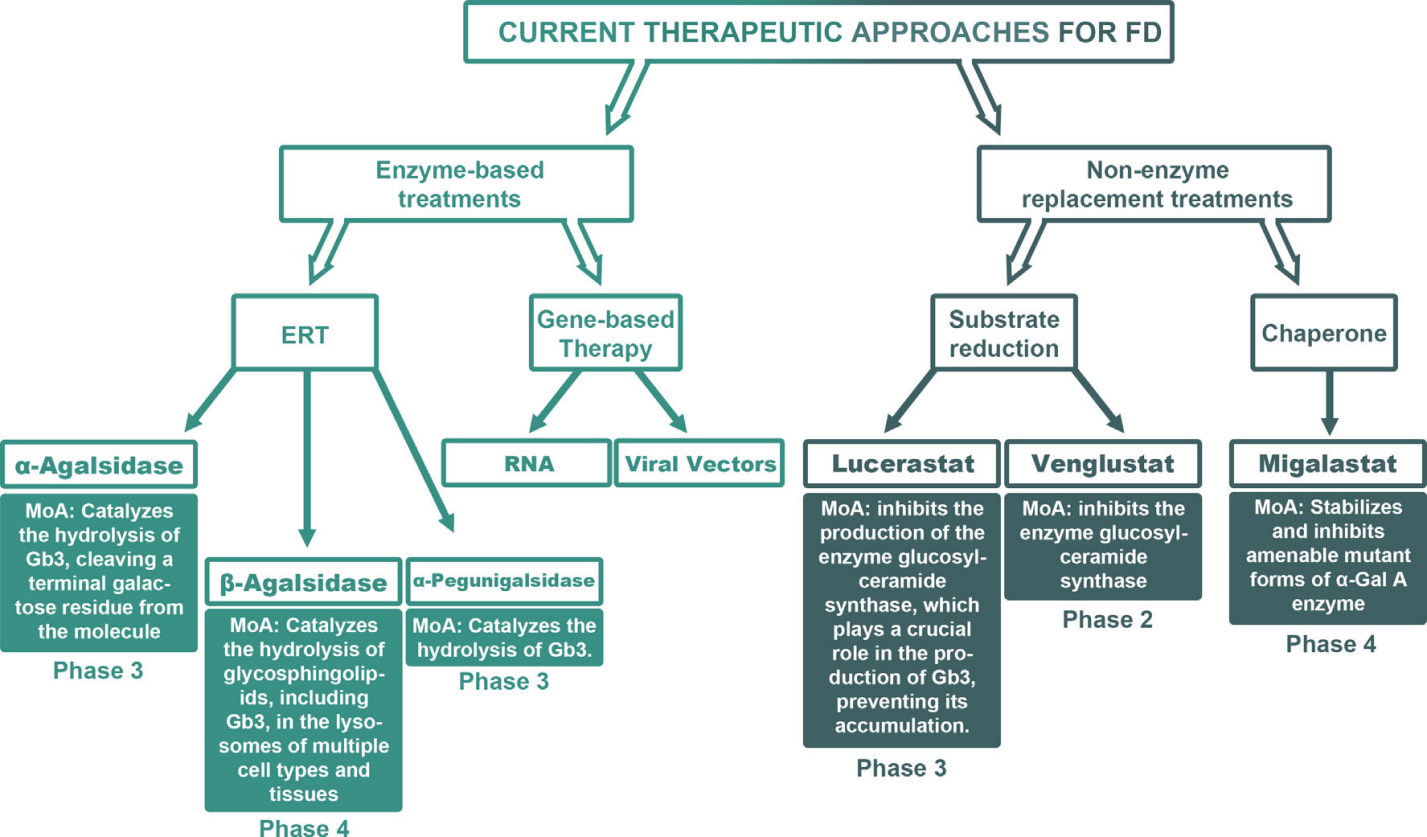
Graph on the current and investigational therapeutic approaches for Fabry disease with the relative mechanism of action (MoA) and the current clinical phase. FD, Fabry disease; ERT, enzyme replacement therapy; RNA, ribonucleic acid

FD therapies are mainly based on intravenous replacement of the altered enzyme using agalsidase α or β. However, recently, therapeutic approaches for some patients affected by FD have been extended (Figure 2).

### 2.1 Enzyme‐based treatments

One of the first therapeutic approach for FD was enzyme replacement therapy (ERT). The treatment successfully improved patient quality of life and alleviating kidney failure, but unmet clinical needs persist.^18^ Ex vivo and in vivo approaches are necessary to increase the enzyme duration in plasma and ameliorate the delivery.^19^

ERT with recombinant α‐galactosidase was approved in Europe in 2001. Two different preparations are accessible: agalsidase α (Replagal®, Shire) produced in a human cell line, is licensed in Europe and other countries and administered at the dose of 0.2 mg/kg; and agalsidase β (Fabrazyme® Sanofi Genzyme), derived from genetically engineered Chinese hamster ovary cells and administered at the licensed dose of 1 mg/kg body weight (intravenous infusion every 2 week). Both treatments are available in most European countries, Australia, in Canada and in Asia.^19^, ^20^ In USA, only Fabrazyme is licensed.

Although ERT has been in clinical use since 2001, the optimal dosage, treatment target and beginning of the treatment need to be elucidate.^1^, ^21^ This is relevant because ERT is costly and is a lifelong obligation for FD patients.^21^ Since it is a rare disorder, it results difficult to analyze the data with meta‐analyses and meta‐syntheses.

It has been demonstrated that short‐term treatment with Replagal reduced plasma Gb3 levels by 50%.^22^ Also, patients on dialysis or who had received a transplant showed comparable level clearance, in particular, plasma Gb3 levels decreased by 43% after 27 weeks of treatment.^23^ Variations in Gb3 levels have been investigated in several pediatric trials.^24^, ^25^, ^26^

In 2001, Schiffmann and collaborators published the first randomized, double‐blind, placebo‐controlled Phase II/III trial with Replagal.^27^ Notably, this trial showed from primary end points significant statistically and clinically amelioration of neuropathic pain. In the secondary end points, the patients showed great improvement in creatinine clearance and cardiac conduction, implying that the therapeutic effects of the drug are prevalent.^27^ Numerous other randomized, controlled trials and open‐label studies validated the safety and efficacy of Replagal.^28^, ^29^, ^30^, ^31^

Clinical trials reported that Fabrazyme induces the clearance of Gb3 in glomerular and mesangial endothelial cells.^19^, ^32^ Additionally, the treatment can reduce pain and critical clinical events also in aged patients.^33^ Furthermore, Fabrazyme treatment was able to ameliorate vascular functions in FD patient from the first infusion and then maintained for the whole period of observation (1 year).^34^

The aspects of ERT are substantially reviewed in.^35^, ^36^, ^37^, ^38^ Overall, Replagal and Fabrazyme share comparable biochemical and structural characteristics, even though some physicians indicated the recommended dosage of Fabrazyme five times greater. The switch to Replagal resulted well tolerated.^39^, ^40^

In contrast, a cohort study reported that Fabrazyme treatment led to improved heart and biochemical amelioration (even in the presence of antibodies) and a greater decrease in plasma lyso‐Gb3 levels in men with classical FD compared to Replagal.^20^ However, the two groups showed no differences in GFR and clinical event rate.^20^

ERT is usually well tolerated, however may appear reactions due to the infusion, which lead to dyspnea, rash or hyperpyrexia and rarely anaphylaxis.^19^, ^41^ Premedication of steroid or diphenhydramine are used to diminish these symptoms.^19^ Moreover, the infusion rate is a key issue (faster rates are linked to higher intolerance) but also the frequency of the infusions. Classical FD male patients should start ERT promptly, with adjunct therapeutic approaches if necessary. Adult women with classic mutation should initiate ERT if the symptoms involve major organs or, if asymptomatic, when the histological, imaging or laboratory investigations reveal damage in the primary organs.^42^

### 2.2 Investigational Exogenous Enzyme Replacement Therapies

Among the novel ERT, α‐pegunigalsidase is a pegylated form of α‐galactosidase generated in a PlantCell Ex system. The use of plant‐based compounds is rapidly increasing in the area of medicine, since it is more efficient at lower costs.^43^In addition, preclinical data showed that the vascular half‐life of plant‐based biologics is higher than existing ERT. It has been reported reduced uptake by the liver and improved uptake by kidney and heart when compared to presently available ERT preparations that are characterized by terminal mannose‐6‐phosphate glycosylation residues.^44^

Existing ERT therapies showed 2 hours of half‐life, while the plant‐based approach showed 53–121 hours of half‐life. Moreover, the drug is well tolerated with mild adverse events.^44^ Notably, during the 9 months of treatment, most of the patients showed a substantial decrease in Gb3 accumulation.^45^

Clinical trials tried to compare this approach to α‐ and β‐galactosidase to understand the potential of pegunigalsidase. An open‐label switchover study is ongoing (NCT03180840), to assess the pharmacokinetics of α‐pegunigalsidase.^46^

### 2.3 Gene‐based therapy

Gene‐based therapy represents a potential approach for different rare genetic disorders, among which FD. For the gene therapy in vivo, a vector is infused into the FD patient and then the cells, including liver cells, promptly undertake gene editing to express the missing protein.^47^

A recent approach is based on the adeno‐associated virus (AAV)–mediated gene transfer infusion in order to enhance the enzyme level. Studies on α‐galactosidase A knockout (*GLA*ko) mouse model demonstrated that this approach was able to induce the production of α‐galactosidase by the liver in a dose‐dependent manner.^48^ A comparable method utilizing another vector was able to strongly increase α‐galactosidase levels after single‐stranded AAV8 vector administration and lyso‐Gb3 and Gb‐3 levels decreased comparable to wild‐type animals.^49^

In the ex vivo method, hematopoietic stem cells from the patient are harvested, underwent gene editing and subsequently are engrafted back to the patient after myeloablative therapy. To date, there are different ex vivo gene therapy approaches for FD treatment.

Huang and collaborators reported that CD34‐positive hematopoietic stem cells, modified using recombinant lentivirus‐mediated gene infused into autologous recipients, showed good engraftment and continuous α‐galactosidase production at 1 and 2 years.^19^ In a phase II trial using ex vivo gene therapy was able to increase *α‐gal A* activity in 2 patients.^50^

Recently, to increase the delivery of human α‐gal protein in vivo nanoparticle‐formulated mRNA in mouse and nonhuman primate were used, with no necessity for myeloablative therapy or viral vectors administration. In particular, the mRNA for h*GLA* was encapsulated with lipid nanoparticles and was able to increase α‐galactosidase expression in cardiac, liver, and kidney tissues, causing increased Gb‐3 clearance.^49^

Gene therapy approaches have the common aim to increase α‐galactosidase enzyme activity. Before adopting gene therapy for FD, sufficient α‐gal A activity and stable viral copy number should be considered compared to current ERT.

### 2.4 Nonenzyme replacement approaches

To reduce Gb3 deposits, nonenzyme replacement approaches are also used and comprise the inhibition of glucosylceramide synthase. Chaperone therapy is able to stabilize and increase the endogenous enzyme activity and is now approved for a specific group of FD mutations. These mutations that produce a form of α ‐Gal A which responds to chaperone binding with a relevant increase in function are known as amenable mutations.

Chemical chaperones are able to attach to the defective enzyme, leading to its proper folding, maturation and trafficking to the functional site.^51^ A recent approved pharmacologic chaperone is the low molecular weight iminosaccharide Migalastat. Migalastat (Galafold™) stabilizes and improves trafficking of amenable mutations of *α‐gal A* enzyme from the endoplasmic reticulum to lysosomes and increases its lysosomal activity.^52^, ^53^

It is administered orally showing a significant distribution and it is able to cross the blood brain barrier. The randomized trial ATTRACT intended to study in FD patients the switch from ERT to Migalastat. Migalastat was able to maintain at a low‐level plasma globotriaosylsphingosine, and, interestingly, induced a significant decrement in left ventricular mass index compared to ERT patients. Regarding side effects, headache, nausea, urinary infection and pyrexia were the most frequent.^54^

In 2018, the Food and Drug Administration approved Migalastat basing on FACETS, a randomized, double‐blind, placebo‐controlled phase III study.^52^ 35 to 50% FD amenable mutations can be treated with Migalastat, however, the in vitro assays reported discordant results in the effectiveness of this treatment for some mutations and additional investigations are needed.^54^ Migalastat is not indicated for use in FD patients with nonamenable *GLA* mutations or with severe renal failure.^55^ Another iminosaccharide used as FD treatment is Lucerastat (Idorsia Pharmaceuticals), which functions as inhibitor, since it is able to avoid Gb3 deposits, reducing the amount of ceramide converted in glycosphingolipid.^56^ Lucerastat is currently under investigation in a phase 3 trial (NCT03425539) in order to evaluate the safety and efficacy in FD patients as oral monotherapy.

Another substrate reduction inhibitor is Venglustat by Sanofi Genzyme; it is under phase 2 clinical trial (NCT02489344) to determine its effectiveness in male FD patients.^19^

### 2.5 Adjunctive Therapies

For FD patients is necessary to also follow the standards of care for patients affected by cardiomyopathy and chronic renal disorder. To contain the renal failure, angiotensin receptor blockers and angiotensin‐converting enzyme inhibitors resulted valid treatment for FD patients, concomitant with a low‐sodium diet.^57^ FD is a risk factor for stroke and statins therapy should be take into account.^58^ A study^59^ reported the effectiveness of ERT and antiproteinuric therapy with ACE inhibitors and/or angiotensin II receptor blockers in patients with severe Fabry nephropathy. Patients showed preservation of kidney function if Fabrazyme treatment was initiated at a younger age, and urine protein to creatinine ratio was maintained at or below 0.5 g/g with both antiproteinuric therapies.^59^

## 3 EXPERIMENTAL MODELS

### 3.1 In vivo models

An animal model of this disorder would be useful to investigate beneficial approaches for patients with FD as well as for the study of the underlying molecular pathophysiology (Table 1).

**TABLE 1:** Summary of the available FD in vivo models

In vivo models	Characteristics	Clinical relevance
*GLA*ko mouse	First model developed in 1997 by disrupting *α‐gal A* gene by homologous recombination^51^	Useful for the advancement of efficient therapeutic approaches for FD patients.
TgG3S mouse	To increase Gb3 levels in mouse organs, they generated a transgenic mouse expressing human α1,4‐galactosyltransferase (Gb3 synthase).^58^	Allow to evaluate the active‐site‐specific chaperone therapy.
TgG3S/*GLA*ko mouse	*GLA*ko mouse was crossbred with transgenic mice TgG3S.^60^	Appropriate for preclinical studies (in particular renal failure).
*GLA*ko rat	This model was developed using CRISPR/Cas9 technology to delete the *GLA* gene,^61^, ^62^, ^63^ to see if larger animals showed clinical symptoms typical of FD patients.	Appropriate for preclinical studies (in particular cardiorenal phenotypes and ocular and hearing problems).

The first model reported was in 1997; Oshima and collaborators developed a mouse model for FD by disrupting α‐gal A gene by homologous recombination, then the mouse *α‐gal A* gene was isolated and characterized.^56^ In particular, *α‐gal A* targeting vector was designed to replace exon 3 and intron 3 of *α‐Gal* gene with a neomycin resistance cassette. The construct was electroporated into 129S4/SvJae‐derived J1 embryonic stem cells. Accurately targeted embryonic stem cells were then inoculated into blastocysts and the resultant chimeric males were bred to C57BL/6 females. These mice showed undetectable α‐Gal enzyme activity in cultured fibroblasts as well as the tissues, thus indicating an effective disruption of the *α‐gal A* gene.^64^

These *α‐gal A*‐deficient mice created by gene targeting are clinically normal at 10 weeks of age but showed globotriaosylceramide deposits and inclusions in the kidneys, closer to the situation that usually occur in clinic. The correction in *α‐gal A*‐deficient fibroblasts, using multi drug resistance retroviruses containing the lacking enzyme, is relevant for translating in vivo the correction of the *α‐gal A* deficiency.^56^, ^64^ Experimental strategies to produce the clinical phenotype in these mice result useful for the advancement of efficient therapeutic approaches for FD patients.

Successively, the same research group used aged Fabry mice (80 weeks old) and they noticed that the animals did not show clinical sign or organ malfunction.^65^

Another research group generated *α‐gal AKO* mouse and no organ malfunction in aged animals was reported.^60^

Other investigations described neuronal glycosphingolipid accumulation and somatosensory phenotypes modified in Fabry mice, thus revealing neuropathic pain. However, these findings are conflicting,^61^, ^62^, ^63^ probably due to the unavailability of wild type control mice that may concur to the divergences. A theory supporting the asymptomatic Fabry animal is that glycosphingolipid profiles differ between humans and mice. Indeed, the level of Gb3, Gb4, lactosylceramide, red blood cell globosides are lower in mice than humans.^65^

Another group established a transgenic mouse (TgG3S) expressing the human α1,4‐galactosyltransferase (Gb3 synthase). The purpose of the study was to elevate Gb3 levels in mouse organs (in particular in the heart), which allow to evaluate the active‐site‐specific chaperone therapy for FD.^66^

Further, it has been generated a symptomatic mouse model by crossbreeding *GLA*ko mice with transgenic mice TgG3S. TgG3S/*GLA*ko mice showed elevated Gb3 levels in the primary organs and progressive renal failure accompanied by the typical clinical conditions. Upon recombinant *α‐gal A* administration, the urine volume and albumin concentration were strongly decreased in TgG3S/*GLA*ko mice. These data reported that Gb3 storage is a leading pathogenic aspect in the symptomatic phenotype of TgG3S/*GLA*ko mice, and that this model may be appropriate for preclinical studies.^67^

Unfortunately, the developed FD mouse models did not shown the typical clinical symptoms, thus another lab established a Fabry rat model using CRISPR/Cas9 technology to delete the *GLA* gene, to see if larger animals showed a phenotype that better recapitulates the FD clinical symptoms, in particular the neuropathic pain.^68^ These rats accumulated glycosphingolipids in all tissues and were completely deficient of *α‐gal A* activity. Notably, they developed neuropathic pain as demonstrated from the altered cation channel and altered N‐glycan processing within the Golgi, likely due to substrate deposits within the membranes of this organelle.^68^ Recently, the same research group demonstrated that Fabry rats showed also ocular manifestations^69^ and cardiorenal phenotypes,^70^ including renal tubule impairment and mitral valve thickening.

Further, the same research team reported hearing problems in Fabry aged rats, but additional studies are ongoing. Overall, this symptomatic rat model is a valuable addition to the current Fabry mouse models.^71^

### 3.2 In vitro models

Complementary in vitro models for testing potential therapeutic approaches and to understand the underlying mechanisms are really helpful (Table 2).

**TABLE 2:** Summary of the available FD in vitro models

In vitro models	Characteristics
Primary cultures of aortic endothelial cells from *α‐gal A*KO mouse	Advantages: Useful to compare the effects of recombinant *α‐gal A* and ethylenedioxyphenyl‐P4; Disadvantages: Reduced enzymatic activity and limited lifespan.^67^
Endothelial cell line from a Fabry hemizygote patient introducing human telomerase reverse transcriptase genes	Advantages: Prolonged lifespan, expression of different key markers of endothelial cells, reduced *α‐gal A* activity compared to primary endothelial cells from normal individuals. Disadvantages: Difficulties in studying clinical feature (i.e., renal failure or cardiac problems).^68^
Human podocyte model, combining RNA interference technology with lentiviral transduction of podocytes	Advantages: Reduced enzymatic activity and deposits of intracellular Gb3, concomitantly with an increase in autophagosomes (deficiency of mTOR kinase activity); Disadvantages: Difficulties in studying clinical feature (i.e., neuronal dysfunction or cardiac problems).^69^
Gene silencing with short‐hairpin RNA to produce a stable knock‐down of AGA in LA‐N‐2(a human neuroblastoma)	Advantages: This model showed a reduction in the release of the neurotransmitter acetylcholine, indicating that may be useful to understand specific neuronal dysfunctions in FD; Disadvantages: Difficulties in studying other clinical features (i.e., renal failure or cardiac problems).^70^
iPSC from peripheral blood mononuclear cells of a young Chinese FD patient, presenting cardiomyopathy	Advantages: The model showed some FD key features, including impaired contractility, cellular hypertrophy and Gb3 deposits; Disadvantages: Difficulties in studying other clinical features (i.e., renal failure or cardiac problems).^71^
iPSC‐cardiomyocytes from other two FD patients	Advantages: GL‐3 accumulates in the lysosomes of these cells, inducing alteration close to cardiac tissue of FD patients; Disadvantages: Difficulties in studying other clinical features (i.e., renal failure or neuronal dysfunction).^72^
Jurkat cells (a T‐lymphoblastic leukemia cell‐line)	Advantages: These cells present low α‐gal‐A activity, thus are valuable for studying the mitochondrial impairment and oxidative stress in FD; Disadvantages: Difficulties in studying clinical features (i.e., renal failure or cardiac problems).^73^

Abbreviations: GL‐3, globotriaosylceramide; Gb3, globotriaosylsphingosine; FD, Fabry disease, α‐gal‐A, α‐galactosidase A.

An in vitro model of FD was developed from *α‐gal A* KO mouse, in particular, primary cultures of aortic endothelial cells were generated, characterized and maintained in culture. Further, these primary cultures were used to compare the effects of recombinant *α‐gal A* and a strong glucosylceramide synthase inhibitor on Gb3 metabolism (ethylenedioxyphenyl‐P4).^72^

The main limit in using primary endothelial cells is the limited lifespan, thus Shen and collaborators produced an endothelial cell line from a Fabry hemizygote patient, introducing human telomerase reverse transcriptase genes. The generated cell line showed prolonged lifespan and express different key markers of endothelial cells, while the activity of *α‐gal A* was strongly decreased compared with primary endothelial cells from normal individuals.^74^

To study the renal damage in FD, it was necessary to develop a human podocyte model, combining ribonucleic acid (RNA) interference technology with lentiviral transduction of podocytes. In an established human podocyte cell line (HEK293T), knockdown of *α‐gal A* expression caused reduced enzymatic activity and deposits of intracellular Gb3. Concomitantly, the Authors reported an increase in autophagosomes, detected by deficiency of mTOR kinase activity (a negative regulator of the autophagic machinery) and elevated level of LC3‐II. This model provides encouraging new directions for additional studies on glomerular injury in Fabry patients.^73^

Another in vitro model was based on gene‐silenced cells, in particular, it has been applied gene silencing with short‐hairpin RNA to produce a stable knock‐down of AGA in LA‐N‐2, a human neuroblastoma that can be differentiated to neuronal‐like cells with cholinergic phenotype. This model showed a reduction in the release of the neurotransmitter acetylcholine, indicating that it may be a useful model to understand specific neuronal functions in FD.^75^

Chou and collaborators generated patient‐specific induced pluripotent stem cells (iPSC) from peripheral blood mononuclear cells of a young Chinese FD patient, presenting cardiomyopathy. Peripheral blood mononuclear cells from a 30‐year old Chinese man with FD, *GLA* gene (IVS4 + 919G > A) mutation were reprogrammed into iPSCs and differentiated into iPSC‐ cardiomyocytes and the energy metabolism was evaluated. The model showed some FD key features, including impaired contractility, cellular hypertrophy and Gb3 deposits.^76^

Earlier, another research group developed iPSC‐cardiomyocytes from other two patients. In these cells, Gb‐3 accumulates in the lysosomes inducing alteration close to cardiac tissue of FD patients. Further, substrate reduction therapy by glucosylceramide synthase inhibition was able to counteract Gb3 storage and to remove lysosomal GL‐3 in cardiomyocytes.^77^ This in vitro model represents a useful tool to understand the cardiovascular underlying mechanism and to detect innovative treatments for FD.

Recently, Birket *et al*., used this in vitro model to study the cardiac‐related molecular and functional consequences of *GLA* mutations. Notably, they reported potential new cellular and secreted protein biomarkers. In example, the increase of LIMP‐2 (large inhibitor of metalloproteinases 2), a lysosomal protein involved in heart disorders, due to *α‐gal A* deficiency, could induce the protein secretion, suggesting its essential role in FD pathology.^78^

Another recent paper reported the use of Jurkat cells, a T‐lymphoblastic leukemia cell‐line, as FD model.^79^ These cells present low α‐gal‐A activity, thus are valuable to evaluate *α‐gal‐A* overexpression in presence or absence of impaired mitochondrial respiratory chain. This group reported mitochondrial dysfunction in FD, thus they postulated that agents able to promote mitochondrial activity could represent promising therapeutic approaches. Moreover, since oxidative stress is a result of loss of respiratory chain complex function in FD, the use of antioxidants could be used as adjuvant therapy.^79^

## 4 CONCLUSION

In the recent years, our understanding and available therapeutic approaches for FD received significant improvements. However, the underlying molecular mechanisms and the effective treatments available for FD patients are still limited. Currently, ERT and chaperone therapies are approved for FD, even though there is no cure, because these approaches are able only to reduce the progression of this disorder. Furthermore, FD is still difficult to diagnose because show clinical features overlapping to other pathologies, including the other lysosomal storage diseases, various systemic and rheumatic and immune‐mediated disorders, such as familial Mediterranean fever and rheumatism.^80^, ^81^ Awareness of FD among clinicians can allow an early diagnosis and thus reduce morbidity and mortality.

It would be interesting for future investigations to study the efficacy of the so far developed therapies in combination (not only as solo agent). Another important point to reexamine is the optimal time to begin a specific therapy considering the family history, the onset, the FD variant and the sex. The current available in vivo and in vitro models are valuable for a better understanding of the pathogenesis and underlying mechanism of FD. These models should be made available for a larger audience in order to study this rare disease, therefore, the available therapeutics can be improved, and new approaches can be established.

## CONFLICT OF INTEREST

The authors declare no conflict of interest.

## FUNDING

No funding source.

### PEER REVIEW

The peer review history for this article is available at https://publons.com/publon/10.1111/cge.13999.

## Data Availability

Data sharing is not applicable to this article as no new data were created or analyzed in this study.

## References

[1] Germain DP. Fabry disease. Orphanet J Rare Dis. 2010;5:30. 10.1186/1750-1172-5-30. 21092187 PMC3009617

[2] Kubo T. Fabry disease and its cardiac involvement. J Gen Fam Med. 2017;18(5):225‐229. 10.1002/jgf2.76. 29264031 PMC5689443

[3] Dupont FO, Gagnon R, Boutin M, Auray‐Blais C. A metabolomic study reveals novel plasma lyso‐Gb3 analogs as Fabry disease biomarkers. Curr Med Chem. 2013;20(2):280‐288. 10.2174/092986713804806685. 23092136

[4] Aerts JM, Groener JE, Kuiper S, et al. Elevated globotriaosylsphingosine is a hallmark of Fabry disease. Proc Natl Acad Sci U S A. 2008;105(8):2812‐2817. 10.1073/pnas.0712309105. 18287059 PMC2268542

[5] Kalliokoski RJ, Kantola I, Kalliokoski KK, et al. The effect of 12‐month enzyme replacement therapy on myocardial perfusion in patients with Fabry disease. J Inherit Metab Dis. 2006;29(1):112‐118. 10.1007/s10545-006-0221-3. 16601877

[6] Kok K, Zwiers KC, Boot RG, Overkleeft HS, Aerts JMFG, Artola M. Fabry Disease: Molecular Basis, Pathophysiology, Diagnostics and Potential Therapeutic Directions. Biomolecules. 2021;11(2):271. 10.3390/biom11020271. 33673160 PMC7918333

[7] Ouyang Y, Chen B, Pan X, et al. Clinical significance of plasma globotriaosylsphingosine levels in Chinese patients with Fabry disease. Exp Ther Med. 2018;15(4):3733‐3742. 10.3892/etm.2018.5889. 29563981 PMC5858121

[8] Liu H‐C, Lin H‐Y, Yang C‐F, et al. Globotriaosylsphingosine (lyso‐Gb3) might not be a reliable marker for monitoring the long‐term therapeutic outcomes of enzyme replacement therapy for late‐onset Fabry patients with the Chinese hotspot mutation (IVS4+919G>A). Orphanet J Rare Dis. 2014;9:111. 10.1186/s13023-014-0111-y. 25047006 PMC4223723

[9] Meng X‐L, Arning E, Wight‐Carter M, et al. Priapism in a Fabry disease mouse model is associated with upregulated penile nNOS and eNOS expression. J Inherit Metab Dis. 2018;41(2):231‐238. 10.1007/s10545-017-0107-6. 29110178

[10] Shu L, Vivekanandan‐Giri A, Pennathur S, et al. Establishing 3‐nitrotyrosine as a biomarker for the vasculopathy of Fabry disease. Kidney Int. 2014;86(1):58‐66. 10.1038/ki.2013.520. 24402087 PMC4077934

[11] Vedder AC, Strijland A, vd Bergh Weerman MA, Florquin S, Aerts JMFG, Hollak CEM. Manifestations of Fabry disease in placental tissue. J Inherit Metab Dis. 2006;29(1):106‐111. 10.1007/s10545-006-0196-0. 16601876

[12] Arends M, Wanner C, Hughes D, et al. Characterization of Classical and Nonclassical Fabry Disease: A Multicenter Study. JASN. 2017;28(5):1631‐1641. 10.1681/ASN.2016090964. 27979989 PMC5407735

[13] Wilcox WR, Oliveira JP, Hopkin RJ, et al. Females with Fabry disease frequently have major organ involvement: lessons from the Fabry Registry. Mol Genet Metab. 2008;93(2):112‐128. 10.1016/j.ymgme.2007.09.013. 18037317

[14] Mehta A, Ricci R, Widmer U, et al. Fabry disease defined: baseline clinical manifestations of 366 patients in the Fabry Outcome Survey. Eur J Clin Invest. 2004;34(3):236‐242. 10.1111/j.1365-2362.2004.01309.x. 15025684

[15] van der Tol L, Smid BE, Poorthuis BJHM, et al. A systematic review on screening for Fabry disease: prevalence of individuals with genetic variants of unknown significance. J Med Genet. 2014;51(1):1‐9. 10.1136/jmedgenet-2013-101857. 23922385

[16] Smid BE, van der Tol L, Biegstraaten M, Linthorst GE, Hollak CEM, Poorthuis BJHM. Plasma globotriaosylsphingosine in relation to phenotypes of Fabry disease. J Med Genet. 2015;52(4):262‐268. 10.1136/jmedgenet-2014-102872. 25596309

[17] Wang RY, Lelis A, Mirocha J, Wilcox WR. Heterozygous Fabry women are not just carriers, but have a significant burden of disease and impaired quality of life. Genet Med. 2007;9(1):34‐45. 10.1097/gim.0b013e31802d8321. 17224688

[18] Komori M, Sakurai Y, Kojima H, Ohashi T, Moriyama H. Long‐Term Effect of Enzyme Replacement Therapy with Fabry Disease. Int J of Otolaryngology. 2013;2013:1‐6. 10.1155/2013/282487. PMC3817706 24223040

[19] Felis A, Whitlow M, Kraus A, Warnock DG, Wallace E. Current and Investigational Therapeutics for Fabry Disease. Kidney Int Rep. 2020;5(4):407‐413. 10.1016/j.ekir.2019.11.013. 32274449 PMC7136345

[20] Arends M, Biegstraaten M, Wanner C, et al. Agalsidase alfa versus agalsidase beta for the treatment of Fabry disease: an international cohort study. J Med Genet. 2018;55(5):351‐358. 10.1136/jmedgenet-2017-104863. 29437868 PMC5931248

[21] Desnick RJ, Schuchman EH. Enzyme Replacement Therapy for Lysosomal Diseases: Lessons from 20 Years of Experience and Remaining Challenges. Annu Rev Genom Hum Genet. 2012;13(1):307‐335. 10.1146/annurev-genom-090711-163739. 22970722

[22] Clarke JTR, West ML, Bultas J, Schiffmann R. The pharmacology of multiple regimens of agalsidase alfa enzyme replacement therapy for Fabry disease. Genet Med. 2007;9(8):504‐509. 10.1097/GIM.0b013e318133fb1b. 17700388

[23] Pastores GM, Boyd E, Crandall K, Whelan A, Piersall L, Barnett N. Safety and pharmacokinetics of agalsidase alfa in patients with Fabry disease and end‐stage renal disease. Nephrol Dial Transplant. 2007;22(7):1920‐1925. 10.1093/ndt/gfm096. 17395657

[24] Ries M, Clarke JT, Whybra C, et al. Enzyme replacement in Fabry disease: pharmacokinetics and pharmacodynamics of agalsidase alpha in children and adolescents. J Clin Pharmacol. 2007;47(10):1222‐1230. 10.1177/0091270007305299. 17698592

[25] Ries M, Clarke JTR, Whybra C, et al. Enzyme‐replacement therapy with agalsidase alfa in children with Fabry disease. Pediatrics. 2006;118(3):924‐932. 10.1542/peds.2005-2895. 16950982

[26] Ramaswami U, Wendt S, Pintos‐Morell G, et al. Enzyme replacement therapy with agalsidase alfa in children with Fabry disease. Acta Paediatr. 2007;96(1):122‐127. 10.1111/j.1651-2227.2007.00029.x. 17187618

[27] Schiffmann R, Kopp JB, Austin HA, et al. Enzyme replacement therapy in Fabry disease: a randomized controlled trial. JAMA. 2001;285(21):2743‐2749. 10.1001/jama.285.21.2743. 11386930

[28] Sasa H, Nagao M, Kino K. Safety and effectiveness of enzyme replacement therapy with agalsidase alfa in patients with Fabry disease: Post‐marketing surveillance in Japan. Mol Genet Metab. 2019;126(4):448‐459. 10.1016/j.ymgme.2019.02.005. 30803893

[29] Schiffmann R, Askari H, Timmons M, et al. Weekly enzyme replacement therapy may slow decline of renal function in patients with Fabry disease who are on long‐term biweekly dosing. J Am Soc Nephrol. 2007;18(5):1576‐1583. 10.1681/ASN.2006111263. 17409308 PMC1978101

[30] Schiffmann R, Ries M, Timmons M, Flaherty JT, Brady RO. Long‐term therapy with agalsidase alfa for Fabry disease: safety and effects on renal function in a home infusion setting. Nephrol Dial Transplant. 2006;21(2):345‐354. 10.1093/ndt/gfi152. 16204287

[31] Hajioff D, Goodwin S, Quiney R, Zuckerman J, MacDermot KD, Mehta A. Hearing improvement in patients with Fabry disease treated with agalsidase alfa. Acta Paediatr Suppl. 2003;92(443):28‐30; discussion 27. 10.1111/j.1651-2227.2003.tb00217.x. 14989462

[32] Eng CM, Guffon N, Wilcox WR, et al. Safety and efficacy of recombinant human alpha‐galactosidase A replacement therapy in Fabry's disease. N Engl J Med. 2001;345(1):9‐16. 10.1056/NEJM200107053450102. 11439963

[33] Ortiz A, Abiose A, Bichet DG, et al. Time to treatment benefit for adult patients with Fabry disease receiving agalsidase β: data from the Fabry Registry. J Med Genet. 2016;53(7):495‐502. 10.1136/jmedgenet-2015-103486. 26993266 PMC4941144

[34] Stamerra CA, De Feo M, Castelli V, et al. Effects of agalsidase‐β administration on vascular function and blood pressure in familial Anderson–Fabry disease Eur J Hum Genet Published online September 18, 2020. doi:10.1038/s41431-020-00721-9, 218, 224 PMC7868353 32948848

[35] Germain DP, Elliott PM, Falissard B, et al. The effect of enzyme replacement therapy on clinical outcomes in male patients with Fabry disease: A systematic literature review by a European panel of experts. Mol Genet Metab Rep. 2019;19:100454. 10.1016/j.ymgmr.2019.100454. 30775256 PMC6365982

[36] Germain DP, Arad M, Burlina A, et al. The effect of enzyme replacement therapy on clinical outcomes in female patients with Fabry disease ‐ A systematic literature review by a European panel of experts. Mol Genet Metab. 2019;126(3):224‐235. 10.1016/j.ymgme.2018.09.007. 30413388

[37] Spada M, Baron R, Elliott PM, et al. The effect of enzyme replacement therapy on clinical outcomes in paediatric patients with Fabry disease ‐ A systematic literature review by a European panel of experts. Mol Genet Metab. 2019;126(3):212‐223. 10.1016/j.ymgme.2018.04.007. 29785937

[38] Elliott PM, Germain DP, Hilz MJ, Spada M, Wanner C, Falissard B. Why systematic literature reviews in Fabry disease should include all published evidence. Eur Jour of Med Gen. 2019;62(10):103702. 10.1016/j.ejmg.2019.103702. 31195166

[39] Ripeau D, Amartino H, Cedrolla M, et al. Switch from agalsidase beta to agalsidase alfa in the enzyme replacement therapy of patients with Fabry disease in Latin America. Medicina (B Aires). 2017;77(3):173‐179. 28643672

[40] Pisani A, Bruzzese D, Sabbatini M, Spinelli L, Imbriaco M, Riccio E. Switch to agalsidase alfa after shortage of agalsidase beta in Fabry disease: a systematic review and meta‐analysis of the literature. Genet Med. 2017;19(3):275‐282. 10.1038/gim.2016.117. 27608175

[41] Pintos‐Morell G, Beck M. Fabry disease in children and the effects of enzyme replacement treatment. Eur J Pediatr. 2009;168(11):1355‐1363. 10.1007/s00431-009-0937-9. 19242721 PMC2745529

[42] Ortiz A, Germain DP, Desnick RJ, et al. Fabry disease revisited: Management and treatment recommendations for adult patients. Mol Gen Metab. 2018;123(4):416‐427. 10.1016/j.ymgme.2018.02.014. 29530533

[43] Chen Q, Davis KR. The potential of plants as a system for the development and production of human biologics. F1000Res. 2016;5:F1000. 10.12688/f1000research.8010.1. PMC4876878 27274814

[44] Kizhner T, Azulay Y, Hainrichson M, et al. Characterization of a chemically modified plant cell culture expressed human α‐Galactosidase‐A enzyme for treatment of Fabry disease. Mol Genet Metab. 2015;114(2):259‐267. 10.1016/j.ymgme.2014.08.002. 25155442

[45] Schiffmann R, Goker‐Alpan O, Holida M, et al. Pegunigalsidase alfa, a novel PEGylated enzyme replacement therapy for Fabry disease, provides sustained plasma concentrations and favorable pharmacodynamics: A 1‐year Phase 1/2 clinical trial. J Inherit Metab Dis. 2019;42(3):534‐544. 10.1002/jimd.12080. 30834538

[46] u.s. national library of medicine . Clinical trial gov. https://clinicaltrials.gov/

[47] Simonetta I, Tuttolomondo A, Di Chiara T, et al. Genetics and Gene Therapy of Anderson‐Fabry Disease. CGT. 2018;18(2):96‐106. 10.2174/1566523218666180404161315. 29618309

[48] Jeyakumar J, Kia A, McIntosh J, et al. Liver‐directed gene therapy corrects Fabry disease in mice. Mol Genet Metab. 2019;126(2):S80. 10.1016/j.ymgme.2018.12.196.

[49] DeRosa F, Smith L, Shen Y, et al. Improved Efficacy in a Fabry Disease Model Using a Systemic mRNA Liver Depot System as Compared to Enzyme Replacement Therapy. Mol Ther. 2019;27(4):878‐889. 10.1016/j.ymthe.2019.03.001. 30879951 PMC6453518

[50] Huston MW, Yasuda M, Pagant S, et al. Liver‐targeted AAV gene therapy vectors produced by a clinical scale manufacturing process result in high, continuous therapeutic levels of enzyme activity and effective substrate reduction in mouse model of Fabry disease. Mol Genet and Metab. 2019;126(2):S77. 10.1016/j.ymgme.2018.12.187.

[51] Lenders M, Stappers F, Niemietz C, et al. Mutation‐specific Fabry disease patient‐derived cell model to evaluate the amenability to chaperone therapy. J Med Genet. 2019;56(8):548‐556. 10.1136/jmedgenet-2019-106005. 31010832

[52] McCafferty EH, Scott LJ. Migalastat: A Review in Fabry Disease. Drugs. 2019;79(5):543‐554. 10.1007/s40265-019-01090-4. 30875019 PMC6647464

[53] Germain DP, Nicholls K, Giugliani R, et al. Efficacy of the pharmacologic chaperone migalastat in a subset of male patients with the classic phenotype of Fabry disease and migalastat‐amenable variants: data from the phase 3 randomized, multicenter, double‐blind clinical trial and extension study. Genet Med. 2019;21(9):1987‐1997. 10.1038/s41436-019-0451-z. 30723321 PMC6752321

[54] Hughes DA, Nicholls K, Shankar SP, et al. Oral pharmacological chaperone migalastat compared with enzyme replacement therapy in Fabry disease: 18‐month results from the randomised phase III ATTRACT study. J Med Genet. 2017;54(4):288‐296. 10.1136/jmedgenet-2016-104178. 27834756 PMC5502308

[55] European Medicines Agency . Migalastat (Galafold): EU summary of product characteristics. https://www.ema.europa.eu/

[56] Ohshima T, Murray GJ, Swaim WD, et al. alpha‐Galactosidase A deficient mice: a model of Fabry disease. Proc Natl Acad Sci U S A. 1997;94(6):2540‐2544. 10.1073/pnas.94.6.2540. 9122231 PMC20124

[57] Warnock DG, Daina E, Remuzzi G, West M. Enzyme Replacement Therapy and Fabry Nephropathy. CJASN. 2010;5(2):371‐378. 10.2215/CJN.06900909. 20007680

[58] Politei JM. Can we use statins to prevent stroke in Fabry disease? J Inherit Metab Dis. 2009;32(4):481‐487. 10.1007/s10545-009-1156-2. 19495571

[59] Warnock DG, Thomas CP, Vujkovac B, et al. Antiproteinuric therapy and Fabry nephropathy: factors associated with preserved kidney function during agalsidase‐beta therapy. J Med Genet. 2015;52(12):860‐866. 10.1136/jmedgenet-2015-103471. 26490103 PMC4717450

[60] Bangari DS, Ashe KM, Desnick RJ, et al. α‐Galactosidase A knockout mice: progressive organ pathology resembles the type 2 later‐onset phenotype of Fabry disease. Am J Pathol. 2015;185(3):651‐665. 10.1016/j.ajpath.2014.11.004. 25553976

[61] Rodrigues LG, Ferraz MJ, Rodrigues D, et al. Neurophysiological, behavioral and morphological abnormalities in the Fabry knockout mice. Neurob Disease. 2009;33(1):48‐56. 10.1016/j.nbd.2008.09.001. 18848893

[62] Lakomá J, Rimondini R, Donadio V, Liguori R, Caprini M. Pain Related Channels Are Differentially Expressed in Neuronal and Non‐Neuronal Cells of Glabrous Skin of Fabry Knockout Male Mice. PLoS ONE. 2014;9(10):e108641. 10.1371/journal.pone.0108641. 25337704 PMC4206276

[63] Lakomá J, Rimondini R, Ferrer Montiel A, Donadio V, Liguori R, Caprini M. Increased expression of Trpv1 in peripheral terminals mediates thermal nociception in Fabry disease mouse model. Mol Pain. 2016;12(63):1744806916663729. 10.1177/1744806916663729. 27531673 PMC5009828

[64] Ohshima T, Murray GJ, Swaim WD, et al. Galactosidase A deficient mice: A model of Fabry disease. Proc Natl Acad Sci. 1997;94(6):2540‐2544. 10.1073/pnas.94.6.2540. 9122231 PMC20124

[65] Ohshima T, Schiffmann R, Murray GJ, et al. Aging accentuates and bone marrow transplantation ameliorates metabolic defects in Fabry disease mice. Proc Natl Acad Sci U S A. 1999;96(11):6423‐6427. 10.1073/pnas.96.11.6423. 10339603 PMC26897

[66] Shiozuka C, Taguchi A, Matsuda J, et al. Increased globotriaosylceramide levels in a transgenic mouse expressing human alpha1,4‐galactosyltransferase and a mouse model for treating Fabry disease. J Biochem. 2011;149(2):161‐170. 10.1093/jb/mvq125. 20961863 PMC3031308

[67] Taguchi A, Maruyama H, Nameta M, et al. A symptomatic Fabry disease mouse model generated by inducing globotriaosylceramide synthesis. Biochem J. 2013;456(3):373‐383. 10.1042/BJ20130825. 24094090 PMC4160053

[68] Miller JJ, Aoki K, Moehring F, et al. Neuropathic pain in a Fabry disease rat model. JCI Insight. 2018;3(6):e99171. 10.1172/jci.insight.99171 PMC5926911 29563343

[69] Miller JJ, Aoki K, Reid CA, Tiemeyer M, Dahms NM, Kassem IS. Rats deficient in α‐galactosidase A develop ocular manifestations of Fabry disease. Sci Rep. 2019;9(1):9392. 10.1038/s41598-019-45837-1. 31253878 PMC6599056

[70] Miller JJ, Aoki K, Mascari CA, et al. α‐Galactosidase A‐deficient rats accumulate glycosphingolipids and develop cardiorenal phenotypes of Fabry disease. FASEB J. 2019;33(1):418‐429. 10.1096/fj.201800771R. 29979634 PMC6629127

[71] Miller JJ, Kanack AJ, Dahms NM. Progress in the understanding and treatment of Fabry disease. Biochim Biophys Acta Gen Subj. 1864;2020(1):129437. 10.1016/j.bbagen.2019.129437. PMC6981246 31526868

[72] Shu L, Murphy HS, Cooling L, Shayman JA. An *In Vitro* Model of Fabry Disease. JASN. 2005;16(9):2636‐2645. 10.1681/ASN.2005040383. 16033856

[73] Liebau MC, Braun F, Höpker K, et al. Dysregulated Autophagy Contributes to Podocyte Damage in Fabry's Disease. Chatziantoniou C, ed. PLoS ONE. 2013;8(5):e63506. doi:10.1371/journal.pone.0063506 23691056 PMC3656911

[74] Shen J‐S, Meng X‐L, Schiffmann R, Brady RO, Kaneski CR. Establishment and characterization of Fabry disease endothelial cells with an extended lifespan. Mol Genet Metab. 2007;92(1–2):137‐144. 10.1016/j.ymgme.2007.06.003. 17644384 PMC2063578

[75] Kaneski CR, Brady RO, Hanover JA, Schueler UH. Development of a model system for neuronal dysfunction in Fabry disease. Mol Genet Metab. 2016;119(1–2):144‐150. 10.1016/j.ymgme.2016.07.010. 27471012 PMC5031533

[76] Chou S‐J, Yu W‐C, Chang Y‐L, et al. Energy utilization of induced pluripotent stem cell‐derived cardiomyocyte in Fabry disease. Int J Card. 2017;232:255‐263. 10.1016/j.ijcard.2017.01.009. 28082092

[77] Itier J‐M, Ret G, Viale S, et al. Effective clearance of GL‐3 in a human iPSC‐derived cardiomyocyte model of Fabry disease. J Inherit Metab Dis. 2014;37(6):1013‐1022. 10.1007/s10545-014-9724-5. 24850378

[78] Birket MJ, Raibaud S, Lettieri M, et al. A Human Stem Cell Model of Fabry Disease Implicates LIMP‐2 Accumulation in Cardiomyocyte Pathology. Stem Cell Reports. 2019;13(2):380‐393. 10.1016/j.stemcr.2019.07.004. 31378672 PMC6700557

[79] Lambert J, Howe S, Rahim A, Burke D, Heales S. Inhibition of Mitochondrial Complex I Impairs Release of α‐Galactosidase by Jurkat Cells. IJMS. 2019;20(18):4349. 10.3390/ijms20184349. PMC6770804 31491876

[80] Zizzo C, Colomba P, Albeggiani G, et al. Misdiagnosis of familial Mediterranean fever in patients with Anderson‐Fabry disease. Clin Genet. 2013;83(6):576‐581. 10.1111/j.1399-0004.2012.01940.x. 22905681

[81] Moiseev S, Karovaikina E, Novikov PI, Ismailova D, Moiseev A, Bulanov N. What rheumatologist should know about Fabry disease. Ann Rheum Dis. 2020;79(6):e71. 10.1136/annrheumdis-2019-215476 31040120

